# HIV-1 Vpu Neutralizes the Antiviral Factor Tetherin/BST-2 by Binding It and Directing Its Beta-TrCP2-Dependent Degradation

**DOI:** 10.1371/journal.ppat.1000574

**Published:** 2009-09-04

**Authors:** Bastien Mangeat, Gustavo Gers-Huber, Martin Lehmann, Madeleine Zufferey, Jeremy Luban, Vincent Piguet

**Affiliations:** 1 Department of Dermatology and Venereology, University Hospitals and Medical School of Geneva, University of Geneva, Switzerland; 2 Department of Microbiology and Molecular Medicine, University Hospitals and Medical School of Geneva, University of Geneva, Switzerland; Northwestern University, United States of America

## Abstract

Host cells impose a broad range of obstacles to the replication of retroviruses. Tetherin (also known as CD317, BST-2 or HM1.24) impedes viral release by retaining newly budded HIV-1 virions on the surface of cells. HIV-1 Vpu efficiently counteracts this restriction. Here, we show that HIV-1 Vpu induces the depletion of tetherin from cells. We demonstrate that this phenomenon correlates with the ability of Vpu to counteract the antiviral activity of both overexpressed and interferon-induced endogenous tetherin. In addition, we show that Vpu co-immunoprecipitates with tetherin and β-TrCP in a tri-molecular complex. This interaction leads to Vpu-mediated proteasomal degradation of tetherin in a β-TrCP2-dependent manner. Accordingly, in conditions where Vpu-β-TrCP2-tetherin interplay was not operative, including cells stably knocked down for β-TrCP2 expression or cells expressing a dominant negative form of β-TrCP, the ability of Vpu to antagonize the antiviral activity of tetherin was severely impaired. Nevertheless, tetherin degradation did not account for the totality of Vpu-mediated counteraction against the antiviral factor, as binding of Vpu to tetherin was sufficient for a partial relief of the restriction. Finally, we show that the mechanism used by Vpu to induce tetherin depletion implicates the cellular ER-associated degradation (ERAD) pathway, which mediates the dislocation of ER membrane proteins into the cytosol for subsequent proteasomal degradation. In conclusion, we show that Vpu interacts with tetherin to direct its β-TrCP2-dependent proteasomal degradation, thereby alleviating the blockade to the release of infectious virions. Identification of tetherin binding to Vpu provides a potential novel target for the development of drugs aimed at inhibiting HIV-1 replication.

## Author Summary

To efficiently replicate in cells, HIV-1 needs to inactivate a number of intracellular host defenses. One such antiviral mechanism is provided by the newly identified tetherin protein. This factor blocks viral production by impeding the release of newly generated HIV-1 particles from the surface of cells. HIV-1 possesses the Vpu protein, which efficiently counteracts this blockade. Here we reveal that HIV-1 Vpu interacts with tetherin and leads to its depletion from cells, possibly through multiple mechanisms, including proteasomal degradation. In order to eliminate tetherin, Vpu hijacks a cellular component, named β-TrCP2, which is normally used by human cells to induce degradation of certain proteins. Identification of tetherin binding to Vpu provides a potential novel target for the development of drugs aimed at inhibiting HIV-1 replication.

## Introduction

In order to successfully infect human cells, HIV-1 has to neutralize cellular restriction factors that impede its replication at multiple steps. HIV-1 Vpu serves this goal by counteracting a blockade imposed by the newly identified protein tetherin [1]–[4]. Under basal conditions, tetherin is expressed in B and T cells, plasmacytoid dendritic cells and myeloid cells [5]–[7]. In addition, tetherin expression is strongly upregulated in many cell types by type-I interferon (IFN), a situation typically encountered in viral infections [5]. Tetherin is a heavily glycosylated type-II transmembrane protein with an unusual topology, which is otherwise only found in mammals in a minor but pathologically important topological variant of the prion protein [8],[9]. Tetherin is indeed linked to membranes both by its one-pass transmembrane domain and by a C-ter GPI anchor. This anti-viral factor is mostly intracellular, but it is also localized at the cell surface in lipid rafts, from where it is continually recycled to the trans-Golgi network [8],[10]. In cells expressing tetherin, HIV-1 viruses deleted for the Vpu gene can bud normally but remain tethered to the cell surface through a protein bond [1],[9]. The mechanistic details of this phenomenon remain to be clarified. A hypothesis, that still awaits confirmation, is that tetherin itself forms the protein tether between the cell surface and the virion owing to its ability to form stable dimers [11]. The affected virions are then endocytosed and probably degraded in lysosomes [1]. In addition to inhibiting HIV-1, tetherin also blocks the replication of numerous retroviruses, as well as other non-related enveloped viruses [12]–[14]. The importance of this restriction in the cellular antiviral arsenal is underscored by the apparent positive selection that tetherin undergoes, which is the hallmark of an ongoing molecular fight with pathogens [15].

Vpu is a transmembrane protein which removes the HIV-1 CD4 receptor from the ER biosynthetic pathway [9]. This is thought to liberate the HIV-1 env glycoprotein from unwanted premature interactions with its receptor. For that task, Vpu bridges CD4 to β-TrCP [16]. β-TrCP (actually representing the two homologues β-TrCP1 and β-TrCP2) is a substrate recognition unit of the SCF (Skp1/Cullin/F-box protein) E3 ubiquitin ligase that provides specificity to this machinery by binding to target proteins harboring a distinct motif (DSGXXS, where both serines are phosphorylated), thereby inducing their ubiquitination and subsequent proteasomal degradation [17],[18]. Vpu possesses a β-TrCP target motif, where the cytosolic serines S52 and S56 are constitutively phosphorylated, which allows efficient recruitment of β-TrCP [19]. Vpu itself escapes degradation by unclear means [16], but instead induces the degradation of the CD4 molecules to which it associates. Of note, the mechanistic details of this action of Vpu are only partly understood, since a direct ubiquitination of CD4 in presence of Vpu is not yet demonstrated [16],[20]. Besides that, Vpu-induced CD4 degradation requires a functional ER-associated degradation pathway (ERAD), which mediates the dislocation of proteins targeted for degradation from ER membranes [21].

Although it had been previously shown that Vpu downmodulates tetherin level from the cell surface [3],[22], the mechanistic details have just begun to be unraveled. It was recently shown that Vpu targets tetherin for proteasomal and/or lysosomal degradation, through a β-TrCP-dependent mechanism [23],[24]. Here we confirm that Vpu leads to a depletion of tetherin from cells. We further show that Vpu performs this action by interacting with tetherin in a ternary complex that also comprises β-TrCP. Importantly, we found this depletion to be functionally relevant since it is required for the efficient counteraction of tetherin-mediated restriction, both in overexpression settings and upon IFN-α-induced endogenous tetherin expression. By generating several cell lines stably knocked-down for β-TrCP1 or β-TrCP2 expression, we further show that β-TrCP2, but not β-TrCP1, is required for this depletion. Furthermore, we confirm that this reduction of tetherin level occurs at least for a large part through the proteasome. The depletion is indeed blocked by a proteasome inhibitor, as well as the K48R mutant of ubiquitin, which allows monoubiquitination of targeted proteins but not the subsequent elongation of the polyubiquitin chains required for proteasomal degradation. In addition, our data are also compatible with a model where some fraction of the Vpu-induced tetherin depletion is due to a β-TrCP2-dependent lysosomal degradation. However, Vpu-induced tetherin degradation explained only a part of its activity against the antiviral factor. Binding of Vpu to tetherin was indeed sufficient for a partial rescue of viral release, even in absence of tetherin degradation. Finally, we show that the mechanism underlying the degradation of tetherin uses a cellular machinery at least partly overlapping with the cellular ERAD pathway.

## Results

### Vpu diminishes cellular levels of human tetherin

**Figure 1:**
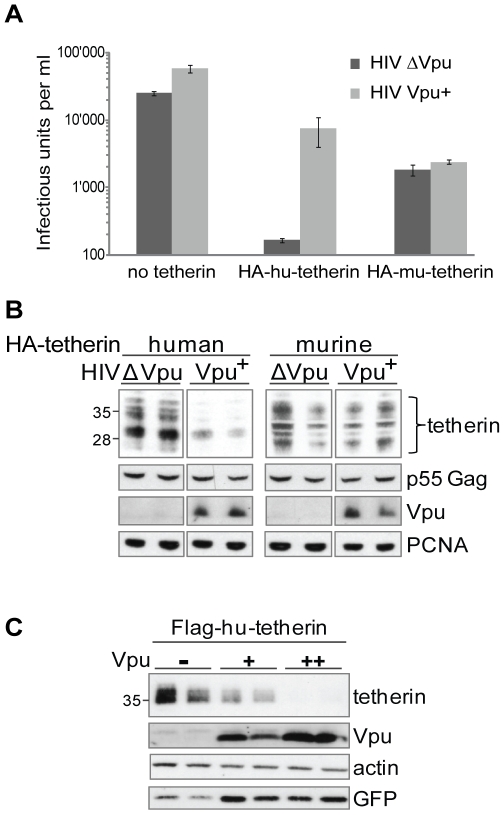
Vpu depletes human tetherin but not murine tetherin, which parallels its ability to rescue virion release. (A) Vpu counteracts human but not murine tetherin antiviral activity. 293T cells were transfected with an HIV-1 provirus either proficient or deficient for the Vpu gene, together with the indicated tetherin constructs. Viral output was then scored by titration of the resulting supernatant on HeLa indicator cells. (B) Vpu depletes human, but not murine, tetherin. Duplicate cell extracts from the above experiment were monitored for tetherin level (as detected with an anti-HA antibody). The viral p55 Gag protein was monitored to exclude variations of transfection efficiency. The depicted tetherin bands correspond to the heterogeneously glycosylated monomer of tetherin of around 30 kDa, but equivalent depletion could be observed for the 60 kDa dimeric form (data not shown). PCNA was used as a loading control. Note that all parts come from the same blot, but the detection of murine tetherin required longer exposure due to its lower expression. (C) Vpu depletes human tetherin in a dose dependent manner, in the absence of other viral components. Increasing doses of a Vpu-expressing plasmid was co-transfected with a Flag-tagged human tetherin in 293T cells (molar ratios of 1∶1 and 2∶1, indicated by + and ++ respectively). Steady state levels of tetherin were monitored by western blot analysis of duplicate cell extracts using an anti-Flag antibody. Transfection levels were assessed with a GFP plasmid, and actin was used as a loading control. All three sections of this figure are representative of five independent experiments performed in duplicate. Sizes of molecular weight markers are shown in kilodaltons.

In order to investigate the mechanistic details of Vpu action against tetherin, we generated constructs of human and mouse tetherin tagged with HA at their cytosolic N-terminus. We expressed these in 293T cells, which do not express endogenous tetherin [2]. In the absence of Vpu, both constructs potently blocked the release of HIV-1 virions as scored by titrating the viral output (Fig. 1A) or by measuring released physical particles by RT assay (data not shown). The lower antiviral activity of murine tetherin is explained by its lower expression level, as indicated by a dose-response assay (data not shown). HIV-1 Vpu expression relieved the blockade imposed by human tetherin, but was only marginally active against murine tetherin, as previously reported (Fig. 1A) [13]. We obtained similar results when the HA tag of tetherin was replaced by a Flag tag (data not shown). This indicates that our system recapitulates the reported restriction imposed by tetherin, at least in its measurable functional consequences. Interestingly, the cellular content of tetherin was markedly reduced in the presence of HIV-1, but not in the presence of the Vpu-deleted version of this virus (Fig. 1B). Paralleling the viral output data, murine tetherin expression levels were not decreased in cells expressing HIV-1 as compared to cells expressing Vpu-deleted HIV-1. Of note, the fact that murine tetherin is not affected by Vpu, although its expression is driven by the same promoter as human tetherin argues against a non-specific transcriptional effect from the viral protein. Additionally, human tetherin depletion was observed when expressed from different unrelated promoters, again arguing against a transcriptional mechanism for Vpu-induced tetherin depletion (data not shown). Finally, we confirmed a very potent and dose-dependent Vpu-mediated human tetherin depletion in cells where HIV-1 Vpu and the antiviral factor are co-expressed (in absence of other viral proteins) (Fig. 1C).

**Figure 2:**
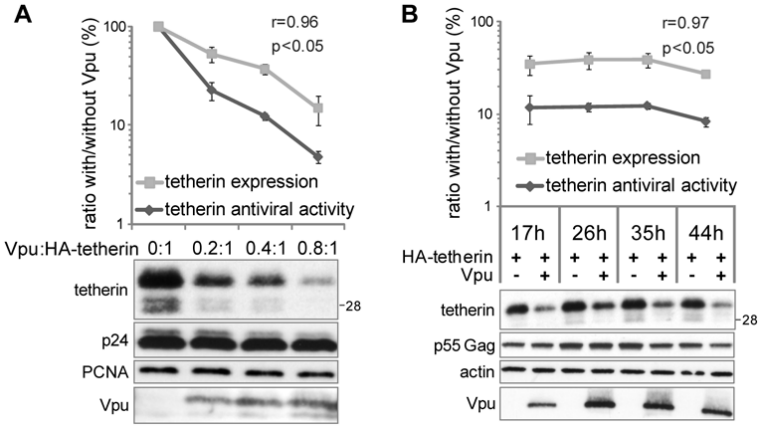
The depletion of tetherin by Vpu correlates with its ability to block tetherin antiviral activity. (A) The depletion of tetherin by Vpu correlates in a dose-dependent manner with its ability to block tetherin antiviral activity. 293T cells were transfected with an HIV-1 provirus deleted for Vpu, together with a fixed dose of human HA-tagged tetherin, and either without or with increasing doses of Vpu added *in trans* (molar ratios are indicated). Viral output was scored by titration of the supernatant on HeLa indicator cells. In parallel, the level of tetherin was monitored by western blotting and subsequently quantified by densitometry. Both the cellular content of tetherin and its antiviral activity were then plotted. The values obtained in the absence of Vpu were given the arbitrary score of 100%. The plot was generated from two independent experiments performed in duplicate. The extracts of duplicate samples were pooled for gel loading. Equal loading was controlled by monitoring PCNA, and the viral p24 protein was examined to exclude variations of transfection efficiency. (B) The depletion of tetherin by Vpu correlates across different time points with its ability to block tetherin antiviral activity. 293T cells were transfected with an HIV-1 provirus deleted for the Vpu gene, with or without a given dose of HA-tagged human tetherin, in the presence or the absence of Vpu added *in trans*. At indicated time points, the titer of the viral output was scored on HeLa indicator cells. In parallel, the level of tetherin was monitored by western blotting and subsequently quantified by densitometry. For each condition, both the cellular content of tetherin and its antiviral activity were plotted as a percent of the values obtained in parallel in the absence of Vpu, which were given the arbitrary score of 100%. The plot was generated from two independent experiments performed in duplicate. The extracts of duplicate samples were pooled for gel loading. Equal loading was controlled by monitoring actin, and the viral p55 Gag protein was examined to exclude variations of transfection efficiency. For both figures, Pearson coefficients of correlation and Student p values were computed for tetherin expression versus antiviral activity. Sizes of molecular weight markers are shown in kilodaltons.

In order to strengthen these observations, we asked whether the Vpu-induced tetherin depletion quantitatively correlated with its ability to rescue viral release. Increasing the dose of Vpu, as expected, proportionally decreased the level of tetherin, which paralleled the decrease in the antiviral activity of the cellular factor (Fig. 2A). The correlation was statistically significant, as indicated by calculating the Pearson coefficient of correlation. Of note, at any given Vpu dose, the decrease of the antiviral activity seemed more efficient than the observed decrease of tetherin level. This most likely reflects depletion-independent activity of Vpu against tetherin. Finally, we determined that the depletion was observed at all tested time points after Vpu and tetherin co-expression (ranging from 17 h to 44 h), and in all these cases, the decrease of tetherin level paralleled the decrease of antiviral activity in a statistically significant manner (Fig. 2B). Overall, these data indicate that Vpu depletes human tetherin from cells in a dose dependent manner, and that this phenomenon is functionally connected to the rescue of viral release exerted by the viral protein.

### Vpu requires its β-TrCP interaction motif to deplete IFN-induced tetherin, and to counteract its antiviral action

**Figure 3:**
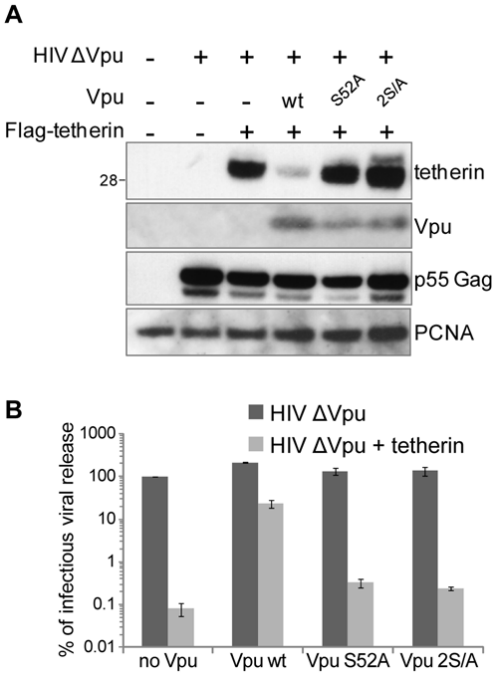
β-TrCP interaction motif is required for Vpu-induced tetherin level reduction and for its ability to rescue virion release. (A) β-TrCP interaction motif is required for Vpu-induced tetherin depletion. HIV-1 deleted for the Vpu gene was produced from 293T cells in the presence or absence of Flag-tagged tetherin. Where indicated, Vpu wild type, or mutated in one (Vpu S52A) or both serines (Vpu 2S/A) known to be required for β-TrCP interaction, was added *in trans*. The effect of these different Vpu constructs on tetherin protein level was monitored by western blotting. The extracts of duplicate samples were pooled for gel loading. Equal loading was controlled by monitoring PCNA, and the viral p55 Gag protein was examined to exclude variations of transfection efficiency. The depicted gel is representative of three independent experiments. Sizes of molecular weight markers are shown in kilodaltons. (B) β-TrCP interaction motif is required for Vpu-induced rescue of virion release. Titer of the viral output obtained during the above experiment was measured on HeLa indicator cells. The titer of the virus produced in the absence of either tetherin or Vpu was given the arbitrary score of 100%. The plot was generated from two independent experiments performed in duplicate.

We wondered whether Vpu depletes tetherin via a mechanism related to its downregulation of CD4. We therefore first asked whether Vpu required an intact β-TrCP interaction motif. For that purpose, we generated a Vpu mutated for one (S52A) or both (S52A and S56A, thereafter coined Vpu 2S/A) of the serines crucial for β-TrCP recruitment [16],[19], and monitored the ability of these constructs to deplete tetherin from transfected 293T cells. Strikingly, both mutants were unable to downregulate tetherin expression (Fig. 3A). Consistent with a crucial role of Vpu-mediated tetherin depletion for HIV-1 replication, we showed that Vpu serine mutants were severely impaired for their ability to counteract tetherin antiviral action (Fig. 3B).

**Figure 4:**
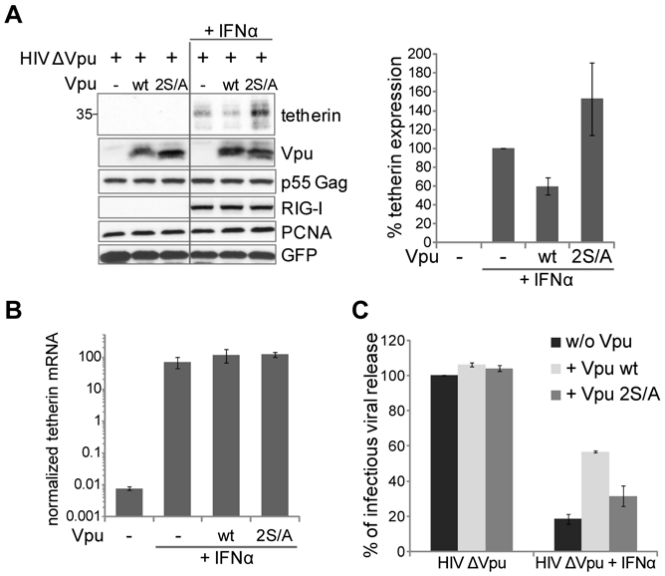
β-TrCP-mediated tetherin degradation is required for Vpu to counteract IFN-α-induced tetherin. (A) β-TrCP interaction motif is required for Vpu-induced depletion of endogenous tetherin. 293T cells were co-transfected with HIV-1 ΔVpu in addition to the indicated Vpu constructs (with a Vpu∶provirus molar ratio of 3∶1). Eighteen hours after transfection, cells were either left untreated or treated for 8 hours with 3000 units per ml of IFN-α to induce tetherin expression and, 20 hours after the end of this treatment, triplicate cell extracts were pooled and analyzed by western blotting to detect endogenous tetherin (left panel). In parallel, RIG-I upregulation was scored to exclude any alteration of IFN receptor signalling by Vpu. Monitoring the viral p55 Gag protein as well as GFP, which was also co-transfected, excluded transfection variations. PCNA served as a loading control. The effect of Vpu constructs on tetherin protein level was quantified by densitometry, with the level of tetherin in the absence of Vpu being given the arbitrary value of 100% (right panel). Sizes of molecular weight markers are shown in kilodaltons. (B) Vpu expression does not affect IFN-α-mediated tetherin mRNA upregulation. Total RNA was extracted and used to monitor tetherin mRNA level by real-time RT-PCR. Expression of the TBP cellular gene was scored in parallel and used as a normalizer. (C) β-TrCP interaction motif is required for Vpu counteraction of the antiviral activity of endogenous tetherin. The viral output obtained at the end of the above experiment was scored by titration on HeLa indicator cells. Titer of the virus produced in the absence of IFN, tetherin or Vpu was given the arbitrary value of 100%. Results from all three panels were generated from two independent experiments performed in duplicate and triplicate, respectively.

We next assessed the importance of β-TrCP recruitment motif of Vpu for tetherin counteraction in cells expressing endogenously the antiviral restriction factor, as opposed to an overexpressed form of the protein. For that purpose, we treated 293T with IFN-α for 8 hours, which potently induced expression of endogenous tetherin both at mRNA and protein levels, as previously reported [2] (Fig. 4A and 4B). In parallel, we transfected these cells with a Vpu-deleted HIV-1 in the absence or presence of wild type Vpu or a Vpu 2S/A mutant. The expression of either Vpu constructs had no significant effects on IFN-receptor signaling as monitored by the induction of RIG-I, a well known IFN-responsive gene (Fig. 4A). Additionally, Vpu or a Vpu 2S/A mutant had no impact on IFN-mediated upregulation of tetherin mRNA (Fig. 4B). Consistent with what we observed in overexpression settings, the cellular content of endogenous tetherin protein was reduced by wild type Vpu expression, but not by its serine mutated counterpart, indicating that Vpu-mediated downregulation of tetherin occurs post-transcriptionally (Fig. 4A). The reduction of endogenous tetherin expression by Vpu in 293T cells treated with IFN-α is more modest than in co-transfection settings, which is expected since here all cells express endogenous IFN-induced tetherin, while only a fraction of these is successfully transfected with Vpu (data not shown). Furthermore, the reduction of endogenous IFN-induced tetherin by Vpu indicates that the depletion observed with tagged versions of the protein does not simply stem from a cleavage of the tag off the tetherin protein. Importantly, the IFN-induced tetherin upregulation led to a defect in viral release for ΔVpu HIV-1, which was rescued when wild type Vpu was added *in trans* (Fig. 4C). Of note, the Vpu-mediated enhancement of viral release was less potent in these settings than upon tetherin overexpression, most probably because IFN treatment induces, apart from tetherin, additional anti-HIV-1 factors that are insensitive to Vpu activity [25]. Correlating with tetherin protein levels, when the double serine mutant of Vpu was used, HIV-1 viral release was only marginally increased. This data with endogenous IFN-induced tetherin confirms the importance of the β-TrCP-recruitment motif of Vpu to counteract tetherin-mediated restriction.

### Vpu requires β-TrCP2 to deplete tetherin from cells and antagonize its antiviral action

**Figure 5:**
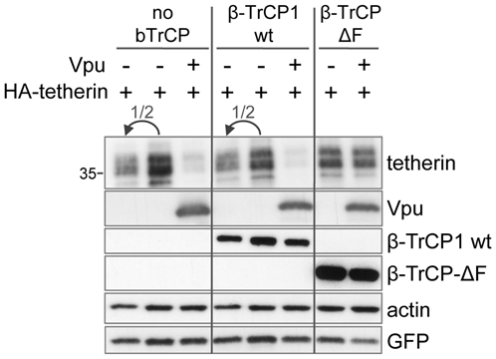
A β-TrCP dominant negative prevents Vpu-mediated tetherin degradation. 293T were transfected with HA-tagged tetherin with or without Vpu, in the presence of either a Flag-tagged dominant negative β-TrCP-ΔF or a Flag-tagged wild type β-TrCP1. The molar ratio of β-TrCP to Vpu to tetherin constructs was 2.5∶2∶1. A GFP plasmid was included to exclude variations in transfection efficiency. The resulting duplicate lysates were pooled for gel loading, and proteins levels were determined by western blotting. Actin served as a loading control. The depicted figure is representative of four independent experiments performed in duplicate. Sizes of molecular weight markers are shown in kilodaltons.

To confirm the involvement of β-TrCP in the anti-tetherin action of Vpu, we tested the effect on Vpu action of a β-TrCP-ΔF deletion mutant, which was shown to abrogate the degradation of CD4 by Vpu [16]. This construct is a well characterized dominant negative of β-TrCP that cannot be anchored on the SCF E3 ligase since it lacks the so-called F-box domain, which mediates β-TrCP binding to the skp1 adaptor of this machinery [17]. This mutant, derived from a β-TrCP1 clone, has dominant negative activity on both β-TrCP1 and β-TrCP2. Strikingly the concomitant expression of this dominant negative form of β-TrCP (β-TrCP-ΔF) completely abolished Vpu-mediated tetherin degradation (Fig. 5). β-TrCP-ΔF expression did not alter significantly Vpu protein levels, as expected. Expression of wild type β-TrCP1 (Fig. 5) and wild type β-TrCP2 did not prevent and even slightly increased Vpu-mediated tetherin downregulation (especially for β-TrCP2) (data not shown). Altogether, these data strongly suggest a role for β-TrCP in the Vpu-mediated counteraction of tetherin.

**Figure 6:**
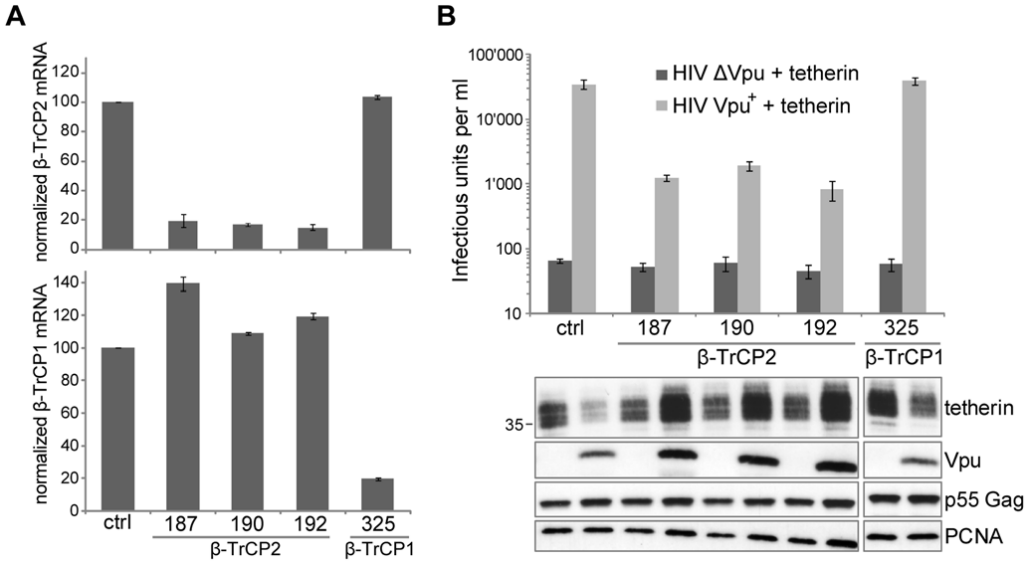
Vpu requires β-TrCP2 to deplete tetherin from cells and antagonize its antiviral action. (A) Creation of 293T cell lines harboring stably downregulated β-TrCP1 and β-TrCP2 levels. Total RNA from 293T cell lines stably expressing the indicated shRNAmir constructs was extracted, and used to monitor β-TrCP1 and β-TrCP2 mRNA levels by real-time RT-PCR. The expression of the TBP cellular gene was used for normalization. The values of β-TrCP1 and β-TrCP2 measured in the presence of the control shRNAmir were given the arbitrary value of 100%. (B) Vpu requires β-TrCP2 to deplete tetherin from cells and antagonize its antiviral action. A Vpu-deleted HIV-1, or its Vpu-proficient counterpart, was transfected in duplicate in the indicated stable cell lines, in the presence of an HA-tagged tetherin plasmid (molar ratio of 2∶1 in favor of tetherin). The extracts of the duplicate samples were pooled for gel loading, and tetherin protein levels were monitored by western blotting (lower panel). Equal loading was controlled by monitoring PCNA, and the viral p55 Gag protein was examined to exclude variations of transfection efficiency. In parallel, titer of the viral output present in the supernatant was monitored on HeLa indicator cells (upper panel). Similar results were obtained by scoring the physical viral particle output by reverse transcription assay (data not shown). The western blot figure is assembled from the data of two gels performed in parallel, on both of which all relevant controls were present and gave identical results. The figure is representative of two independent experiments performed in duplicate. Sizes of molecular weight markers are shown in kilodaltons.

In order to further analyze the requirement for β-TrCP in Vpu anti-tetherin action, we generated 293T cell lines stably transduced with lentiviral vectors expressing microRNA-adapted shRNA (shRNAmir) specifically targeting β-TrCP1 or β-TrCP2. We obtained one cell line harboring potent β-TrCP1 downregulation (shRNAmir #325), and three cell lines harboring potent β-TrCP2 downregulation (shRNAmir # 187, 190 & 192), as measured by real-time RT-PCR (Fig. 6A). In cells that expressed a control or β-TrCP1-targeting shRNAmir, Vpu depleted tetherin very efficiently (Fig. 6B, lower panel). In contrast, in all three cell lines that harbored diminished levels of β-TrCP2, Vpu-induced tetherin depletion was abolished. Importantly, measuring the effect of Vpu on release of HIV-1 in these different cell lines showed a complete correlation between the ability of Vpu to trigger tetherin depletion and its ability to functionally antagonize the antiviral factor (Fig. 6B, upper panel). Of note, Vpu still exhibited a residual activity to rescue viral release in cells depleted for β-TrCP2. Overall, our data demonstrated that Vpu induced tetherin depletion in a β-TrCP2-dependent manner. Finally, in situations where Vpu did not lead to tetherin degradation (Vpu mutants or β-TrCP2 downregulation), we consistently observed that tetherin levels were increased to varying extents above basal levels (Fig. 3A, 4A, 6B). This suggests that Vpu might stabilize tetherin when it is unable to target it to the degradative machinery, possibly via a direct interaction with the protein.

### Vpu and β-TrCP co-immunoprecipitate with tetherin

**Figure 7:**
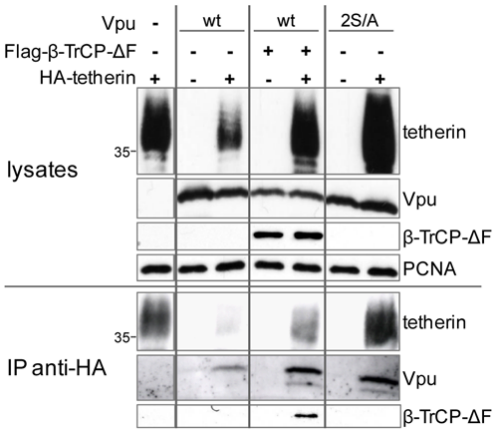
Vpu and β-TrCP co-immunoprecipitate with tetherin. 293T cells were transfected with the indicated Vpu and β-TrCP-ΔF constructs, in the presence or absence of HA-tetherin (with a molar ratio of 2∶1 in the favor of Vpu). Equal amounts of lysates were subjected to immunoprecipitation with an anti-HA resin and analyzed by western blotting. PCNA served as a loading control. The first left lane was cut and pasted from another position from the same scan of the same blot. The figure is representative of two independent experiments. Sizes of molecular weight markers are shown in kilodaltons.

In order to analyze whether Vpu could interact in eukaryotic cells with the antiviral factor, we transfected 293T cells with Vpu in the presence or absence of HA-tagged tetherin. Monitoring the lysates from these co-transfections confirmed Vpu-induced depletion of tetherin (Fig. 7, upper part, lanes 1 and 3). By subsequently immunoprecipitating HA-tetherin with an anti-HA resin, we could show that Vpu was efficiently pulled down in the presence but not in the absence of tetherin (Fig. 7, lanes 1, 2 and 3). In addition, the dominant negative form of β-TrCP (which binds to Vpu, but is unable to recruit the E3 ligase machinery) also co-immunoprecipitated with tetherin. This demonstrates that a ternary complex exists between tetherin, Vpu and β-TrCP. Nevertheless, this experiment does not rule out the possibility that β-TrCP interacts with tetherin also in the absence of Vpu, although this seems unlikely as tetherin itself does not harbor a bone fide β-TrCP recruitment motif. Notably, the tetherin-Vpu interaction was easier to detect in the presence of the β-TrCP dominant negative (ΔF-box) (lanes 4 and 5) or when a Vpu defective for β-TrCP recruitment (Vpu 2S/A) was used instead of wild type Vpu (lanes 6 and 7). This apparent increase in co-immunoprecipitation of tetherin-Vpu complexes might reflect a more stable association between Vpu and tetherin in conditions where the complex cannot be targeted to degradation, or alternatively simply results from higher levels of HA-tetherin present in these extracts, since in these conditions Vpu is not able to reduce tetherin cellular levels (upper part, lanes 4 to 7). These results demonstrate that the inability of Vpu 2S/A mutant to induce tetherin depletion does not stem from an inability to interact with tetherin, but rather originates from its inability to recruit β-TrCP. Finally, this data indicates that binding of Vpu to tetherin is not sufficient to induce its degradation or to fully counteract its antiviral activity, since the Vpu 2S/A mutant strongly binds to tetherin but is significantly impaired for both these activities.

### Vpu requires a functional polyubiquitin/proteasome system for efficient tetherin depletion

**Figure 8:**
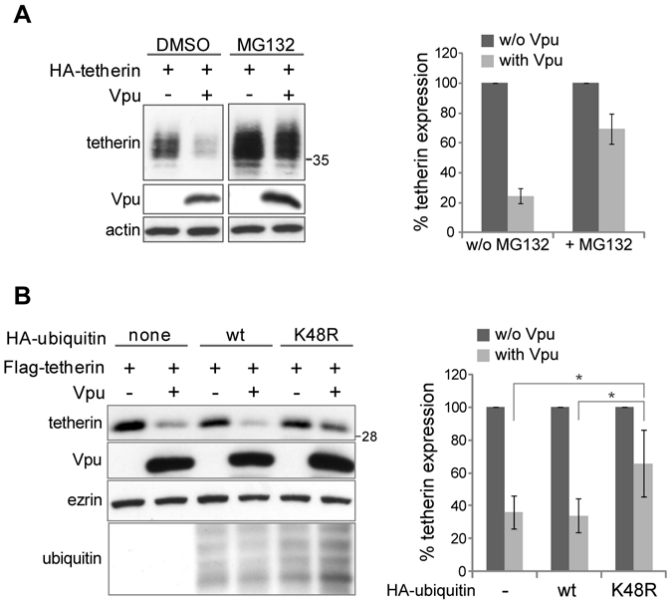
Vpu induces proteasomal degradation of tetherin. (A) The MG132 proteasomal inhibitor impedes Vpu-mediated depletion of tetherin. 293T cells were transfected in duplicate with an HA-tetherin construct in the presence or absence of Vpu (with a molar ratio of 2∶1 in favor of Vpu), and were either left untreated or treated for 12 hours with the proteasome inhibitor MG132. Duplicate lysates were then pooled for western blot analysis (left panel). Actin served as a loading control. The effect of MG132 on Vpu-mediated tetherin depletion was quantified by densitometry and a plot was generated from the results of three independent experiments performed in duplicate (right panel). The values obtained in the absence of Vpu were given the arbitrary value of 100%. (B) An ubiquitin mutant that blocks the formation of the polyubiquitin chains involved in proteasomal targeting impedes Vpu-mediated depletion of tetherin. 293T cells were transfected in duplicate with a Flag-tetherin construct in the presence or absence of Vpu. In addition, cells were co-transfected with a wild type or K48R mutant version of HA-tagged ubiquitin. The molar ratio of ubiquitin to Vpu to tetherin constructs was 2.5∶1.75∶1. Duplicate extracts from these cells were pooled and analyzed by western blotting (left panel). Ezrin was used as a loading control. The effect of the different ubiquitin constructs on Vpu-mediated tetherin depletion was quantified by densitometry, and a plot was generated from the results of three independent experiments performed in duplicate (right panel). The values obtained in the absence of Vpu were given the arbitrary value of 100%. Results were statistically significant as the p value, determined by the Student test, was lower than 0.05 for indicated pairs (*). Sizes of molecular weight markers are shown in kilodaltons.

Our results point out towards a model where Vpu bridges tetherin to β-TrCP2, which leads to the depletion of tetherin from cells and, as a consequence, alleviates the restriction imposed by the antiviral factor. In order to define if Vpu-tetherin-β-TrCP2 complexes were targeted to proteasomal degradation, we transfected 293T cells with an HA-tetherin construct in the presence or absence of Vpu. Forty hours later, the cells were either left untreated or treated with the proteasome inhibitor MG132 for 12 hours and then lysed. This revealed that proteasomal inhibition significantly rescued tetherin expression in presence of Vpu (Fig. 8A). A modest increase of tetherin expression was also noted in the absence of Vpu. As a control, MG132 stabilized the Vpu-resistant murine tetherin to an equal extent in the absence or presence of Vpu (data not shown). Overall, this indicated that Vpu, at least in part, targets tetherin for proteasomal degradation. To confirm this finding, we performed a Vpu and Flag-tetherin co-transfection, with the additional inclusion of wild type ubiquitin or its mutated K48R form, which blocks the formation of the polyubiquitin chains implicated in proteasomal targeting. This construct indeed can be attached to target proteins as a monomer, but due to the absence of the proper acceptor lysine 48, impedes further covalent attachment of additional ubiquitin to the nascent chain [26]. Notably, while wild type ubiquitin had no impact on tetherin depletion, the K48R ubiquitin mutant partially blocked Vpu-mediated tetherin downregulation (Fig. 8B). Finally, we were unable to directly detect ubiquitinated forms of tetherin in presence or absence of Vpu even after treatment with MG132, either because tetherin is not directly ubiquitinated, or because of technical limitations (data not shown). Altogether, these data indicate that Vpu binds to tetherin and concomitantly recruits β-TrCP2 to trigger the proteasomal degradation of the antiviral factor.

### A functional ERAD pathway is required for Vpu-induced tetherin degradation

**Figure 9:**
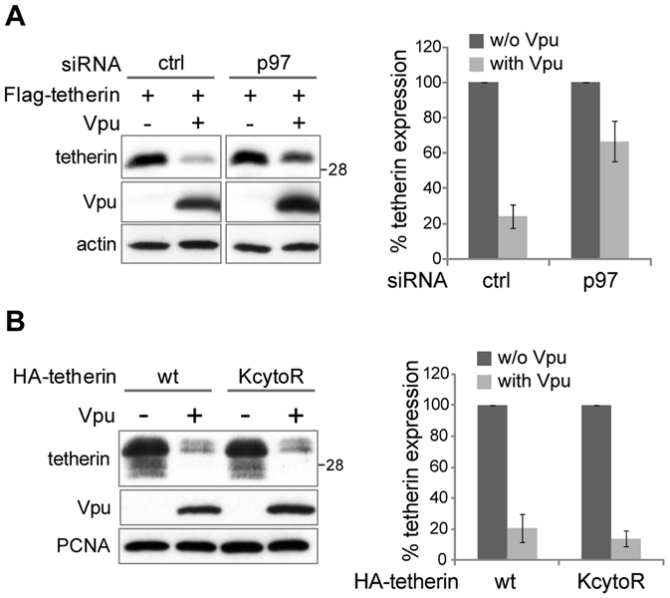
Vpu-induced proteasomal degradation of tetherin involves ERAD. (A) The ERAD pathway is involved in Vpu-mediated tetherin depletion. 293T cells were transfected in duplicate with 100 nM of either a non-silencing control siRNA, or of a siRNA pool targeting p97. Twenty-four hours later, these cells were transfected with Flag-tagged tetherin in the presence or absence of Vpu (with a molar ratio of 2∶1 in favor of Vpu). Duplicate cell lysates were pooled for western blot analysis (left panel). Actin served as a loading control. Note that both parts of the figure come from the same scan of the same blot. The effect of the different siRNAs on Vpu-mediated tetherin depletion was quantified by densitometry and a plot was generated from the results of two independent experiments performed in duplicate (right panel). The values obtained in the absence of Vpu were given the arbitrary value of 100%. (B) Vpu-mediated tetherin depletion does not require ubiquitination of tetherin cytosolic lysines. 293T cells were co-transfected in duplicate with or without Vpu in the presence of either HA-tagged wild type tetherin or its counterpart having both its cytosolic lysines K18 and K21 replaced with arginines (KcytoR tetherin). Duplicate extracts were pooled and analyzed by western blotting. PCNA served as a loading control. Vpu-mediated tetherin depletion was quantified by densitometry, and a plot was generated from the results of two independent experiments performed in duplicate (right panel). The values obtained in the absence of Vpu were given the arbitrary value of 100%. Sizes of molecular weight markers are shown in kilodaltons.

The proteasomal degradation of trans-membrane proteins such as tetherin requires that the cell employs a specific machinery. Indeed, such proteins must be dislocated from membranes prior to their entry into the cytosolic proteasome complex [27]. In the ER, this dislocation is mediated by a series of distinct mechanisms, collectively known as the ERAD (ER-associated degradation) pathways. Briefly, the targeted protein is marked for degradation by a mostly unclear mechanism, which can include ubiquitination. The subsequent dislocation from the membrane is performed by a series of protein complexes which all require at some point the mechanical pulling force generated by the p97 ATPase (also known as VCP). The dislocated protein is then targeted to proteasomal degradation by ubiquitination [27]. To address whether the ERAD pathway is required for Vpu-induced tetherin proteasomal degradation, we transfected 293T with a control siRNA or a siRNA pool specific for p97, which led to a 50% downregulation of its mRNA level (data not shown). This relatively low level of downregulation might be due to the constitutively very high expression of p97 [28]. Nevertheless, co-transfection of these cells with a Flag-tetherin plasmid in the absence or presence of Vpu revealed that p97 downregulation partially impaired Vpu-mediated tetherin degradation (Fig. 9A). Interestingly, the involvement of a dislocation out of the ER membrane in Vpu-mediated tetherin degradation potentially exposes to the cytosolic milieu lumenal lysines that therefore also can serve as ubiquitin acceptors. This might explain our observation that a tetherin mutant with its two cytosolic lysines replaced by arginines (KcytoR) was still efficiently targeted for degradation by Vpu (Fig. 9B).

## Discussion

It has been known for a long time that HIV-1 deleted for the Vpu gene cannot be released efficiently from specific cell types such as macrophages or T cells [29],[30]. The recent identification of the IFN-α induced restriction factor tetherin provides an explanation for this phenomenon [2],[3]. Tetherin impedes release of newly budded virions and mediates their internalization, probably thereby targeting them for lysosomal degradation. Vpu efficiently counteracts this antiviral activity by a mechanism whose details only begin to be revealed [2],[3]. Indeed, while it had been known from some time that Vpu downmodulates tetherin from the cell surface [3],[22], it was only recently shown that Vpu targets tetherin for proteasomal and/or lysosomal degradation [23],[24].

We confirm here that Vpu expression indeed induces a sharp reduction of tetherin protein levels in cells (Fig. 1). This depletion strongly correlated with the extent of inhibition of tetherin antiviral action (Fig. 2). In addition, both the effects of Vpu on tetherin level and antiviral activity were dependent on the recruitment by Vpu of β-TrCP, a substrate recognition subunit of the SCF E3 ubiquitin ligase. Indeed, a Vpu mutant defective for β-TrCP recruitment was impaired for both these activities (Fig. 3). Importantly we also showed, by co-immunoprecipitation studies in eukaryotic cells, that Vpu interacts with tetherin, thereby forming a ternary complex with β-TrCP (Fig. 7). As a confirmation of the importance of the Vpu-β-TrCP interplay, Vpu anti-tetherin action was dependent on the presence of a functional β-TrCP2, as determined by RNA interference studies and by using a β-TrCP dominant negative (Fig. 5 & 6). Of note, while preparing this work for publication, the specific involvement of β-TrCP2 in Vpu-mediated tetherin degradation was reported, as well as the interaction between Vpu and tetherin by co-immunoprecipitation [31],[32]. Remarkably, we did not detect any role for the related protein β-TrCP1. While this might appear surprising in regard to the high similarity between β-TrCP1 and β-TrCP2, these proteins display a significant difference, as the first is nuclear, while the second is cytosolic [33]–[35]. Moreover, a differential functional activity of the two proteins has already been reported, for instance in their ability to induce the degradation of IκB [33]. An alternative explanation to our data could be that β-TrCP1 is not functional in 293T cells, where the experiments were performed. While unlikely, this may have gone unnoticed, as the majority of previous RNA interference experiments against β-TrCP were done with siRNAs targeting simultaneously both β-TrCP1 and β-TrCP2. Importantly, we also demonstrated that Vpu requires β-TrCP-dependent tetherin degradation to antagonize the antiviral activity of endogenous IFN-induced tetherin (Fig. 4), in addition to overexpressed forms of the cellular restriction factor. IFN-induced tetherin is particularly relevant since it mimics conditions likely found during HIV-1 infection. For instance the recent article by Li et al. [36] demonstrates that during the early events of HIV-1 infection, plasmacytoid dendritic cells are attracted to the sites of initial infection in mucosal tissues where they secrete high amounts of IFN-α.

We also showed that efficient Vpu-induced tetherin depletion required a functional proteasomal pathway (Fig. 8). Nevertheless, in addition to Vpu-induced tetherin proteasomal degradation, our results do not exclude additional mechanisms of depletion. Indeed, the proteasomal inhibitor MG132, as well as a dominant negative ubiquitin K48R mutant, only showed a partial inhibition of tetherin degradation (Fig. 8). Of note, a role for lysosomal targeting in Vpu-mediated tetherin counteraction was recently proposed [24],[31]. Our preliminary data indicated that, when using the lysosomal inhibitor bafilomycin and the ubiquitin K63R mutant, which dominantly inhibits the formation of K63-dependent polyubiquitin chains, we observed a partial rescue of tetherin degradation (data not shown). Nevertheless, these results were complex to interpret since lysosomal inhibition also markedly altered the basal level of tetherin in the absence of Vpu, suggesting an important role of lysosomes in the normal trafficking of tetherin. Therefore it is possible that β-TrCP might not only trigger proteasomal degradation of tetherin, but also its lysosomal targeting through monoubiquitination or through the formation of non-conventional chains of ubiquitin linked together on their lysine 63 instead of lysine 48, which is known to target proteins towards lysosomes [37]. In summary, β-TrCP recruitment to the Vpu-tetherin complex could lead to different fates (lysosomal or proteasomal degradation) depending on the cellular compartment where it occurs (as discussed in [38] for other proteins). A dislocation step followed by proteasomal degradation might occur predominantly during the tetherin biosynthesis pathway, while a rerouting of tetherin towards lysosomes might be relatively more important during constitutive tetherin endocytosis and recycling from the plasma membrane [10]. Alternatively, ubiquitination of tetherin could first target it to the lysosome and, subsequently, the remaining cytosolic tail of tetherin could be degraded by the proteasome, as described for the erythropoietin receptor [39]. Finally, it is likely that the relative contribution of proteasomal versus lysosomal degradation varies depending on the cell type used. Indeed, while Vpu-induced lysosomal degradation of tetherin was clearly demonstrated in HeLa cells [24],[31], two other publications showed mostly proteasomal degradation in 293T cells [23],[40]. In any case, these distinct trafficking pathways ultimately result in the absence of tetherin from the cell surface, thereby allowing for the unimpeded release of new viral particles.

The mechanism underlying tetherin degradation had common characteristics with the cellular ER-associated degradation pathway (ERAD), where ER-associated proteins are dislocated and subsequently degraded by the proteasome in the cytosol (Fig. 9A). Indeed Vpu-mediated tetherin degradation required the action of the cellular p97 ATPase, which is a key component of the ERAD [27]. The involvement of an ERAD-like pathway in Vpu anti-tetherin functioning provides an explanation for our observation that a tetherin mutant devoid of cytosolic lysines is still degraded by Vpu as efficiently as wild type tetherin (Fig. 9B). It is indeed extensively documented that ERAD substrates do not require cytosolic lysines for their proteasomal degradation [41]–[44]. Notably, this is also true for Vpu-induced ERAD-mediated CD4 degradation [21]. Two possibilities can explain this apparent paradox. Firstly, lumenal lysines are exposed to the cytosolic milieu during the dislocation step, therefore alleviating the requirement for cytosolic lysines for ubiquitination of the target protein. Secondly, ubiquitination of ERAD substrates can occur on non-lysine residues [45],[46]. The precise timing of ubiquitination and dislocation during ERAD, as well as their functional relationship is not yet fully understood [27]. Nevertheless, we show that a putative β-TrCP-mediated tetherin ubiquitination on cytosolic lysines cannot be the trigger for its dislocation, since in that case the lysine mutant would be resistant to Vpu action. Our results are compatible with a model where Vpu induces the dislocation (be it partial) of tetherin from the membrane, thereby exposing additional lysines for ubiquitination by β-TrCP. Finally, we were unable to detect direct tetherin ubiquitination in the presence of Vpu (data not shown). This is not surprising as several groups have been unable to detect Vpu-induced CD4 ubiquitination although it is generally accepted that Vpu also degrades CD4 through a proteasomal pathway [16],[20]. This failure to detect a membrane protein ubiquitination is not limited to Vpu targets, since the same holds true for the ERAD-mediated MHC-1 degradation induced by the CMV protein US11 [47], where the proteasomal degradation of MHC-I does not seem to be coupled to direct MHC-I ubiquitination, but possibly to the ubiquitination of another associated protein. Finally, overall, the mechanism of action employed by Vpu to counter tetherin restriction shares some similarities with the mechanisms used by Vpu to induce CD4 degradation [21],[48]. This is maybe not surprising, since both these cellular proteins are membrane-associated proteins that impede efficient release of viral particles.

Importantly, our data also suggest that the Vpu anti-tetherin activity is not fully explained by Vpu-mediated tetherin depletion. Indeed, this phenomenon accounted for a large part but not the integrality of the Vpu anti-tetherin functional effect. In particular, in conditions where degradation was completely abrogated, Vpu still had a residual ability to counteract tetherin antiviral action (Fig. 3B, 4C and 6B). In addition, the extent of inhibition of tetherin antiviral activity by Vpu was higher than the decrease of tetherin levels it induced (Fig. 2A & 2B). All these observations are fully consistent with reports indicating that a Vpu mutated in its cytosolic serine motif still possesses a residual ability to rescue viral release [3],[49],[50]. This β-TrCP-independent effect is probably mediated by the Vpu transmembrane domain, since this region by itself harbors some potential for the rescue of viral release [2],[49],[51]. In agreement, we showed that a Vpu mutant impaired for β-TrCP binding was still fully able to interact with tetherin (Fig. 7). It is therefore likely that the residual β-TrCP-independent anti-tetherin activity of Vpu is mediated by the interaction between Vpu and tetherin, which likely happens through their respective transmembrane domains. Accordingly, it was recently shown that modifying tetherin transmembrane region can render it resistant to Vpu counterstrike [15],[40]. This binding might partially impair tetherin antiviral activity, possibly by steric hindrance, or by inducing its downregulation from the cell surface. Vpu would subsequently target tetherin for proteasomal and likely also lysosomal degradation, thereby deploying the integrality of its activity [23],[24],[31],[40]. Of note, during the preparation of this manuscript, it was reported that Vpu could relieve the blockade of viral release even in certain cells lines where it failed to induce tetherin depletion [52]. It can be envisioned that, in these cell types, Vpu would counteract tetherin through its β-TrCP-independent activity. In agreement with this model, Vpu mediated its virion release enhancement, which appeared to be only modest, independently of its β-TrCP-interacting motif in these cells [52].

Hijacking of ubiquitin E3 ligases appears to be a common theme for HIV-1 accessory proteins to counteract host cell restriction factors. In addition to Vpu that serves as a bridge between the E3 β-TrCP substrate recognition module and the targeted restriction factor, HIV-1 Vif developed a similar but slightly different strategy, where it directly replaces the substrate recognition module to induce the degradation of the APOBEC3G antiviral protein [53]. More generally, it will be worth investigating the strategies used by other classes of viruses to counteract the broad antiviral action of tetherin. To conclude, we propose that the molecular interplays revealed here pave the way for the development of new therapeutic strategies targeting the Vpu-tetherin interaction in order to thwart HIV-1 replication.

## Materials and Methods

### Plasmids, reagents

Expression plasmids for untagged tetherin of human and murine origin were obtained from Origene (Rockville, MD). Tetherin was subsequently sub-cloned using standard molecular biology procedures into pCDNA3.1(+) or pEF1 backbones (both from Invitrogen), with either a Flag or HA tag added in frame at their N-terminus. The tetherin mutant harboring lysines to arginines changes in its two cytosolic lysines (K18R and K21R, which we named KcytoR), was engineered with the help of the QuickChange mutagenesis system (Stratagene). Of note, the N-terminal HA-tag appended to this construct does not itself encode for any lysine. The expression plasmid for Vpu, pCDNA-Vphu, encodes a well characterized codon-optimized version of Vpu (made by K. Strebel and S. Bour, obtained through the NIH AIDS Research and Reference Reagent Program, Division of AIDS, NIAID, NIH) [54]. Versions of this plasmid harboring the S52A or S52A/S56A (which we named 2S/A in the core of the text) were made with the help of the QuickChange mutagenesis system (Stratagene). The Vpu-deficient or proficient HIV-1 expression vectors are kind gifts of Didier Trono and are based on the pR9 proviral construct [55]. Wild type β-TrCP1 was expressed from the pCR3-Flag-β-TrCP1 plasmid (a kind gift of Sylvia Rothenberger) [56]. β-TrCP1-ΔF-box was expressed from the pCMV2-FLAG-β-TrCP1-ΔF plasmid (a kind gift or Yinon Ben Neriah) [33]. Wild type β-TrCP1 was expressed from the pCDNA3-β-TrCP2-HA plasmid [57]. HA-tagged wild type and K48R mutant of ubiquitin were expressed from the pRK5 backbone and were obtained from Ted Dawson's lab through Addgene [58]. The K63R version of this ubiquitin construct was engineered with the help of the QuickChange mutagenesis system (Stratagene). The GFP expression plasmid was pEGFP.N1 (Clontech). Bafilomycin A1 (Sigma) was used at a concentration of 50 nM, and MG132 (Sigma) at a concentration of 10 uM. Recombinant human IFN-α was obtained from Sigma.

### Cells and transfections

293T cells were cultured following usual procedures. The transfection of these cells was performed either following a standard calcium-phosphate-based technique or with the help of the Fugene 6 reagent (Roche), according to manufacturer instructions. For experiments done in the absence of proviral constructs, the molar ratio of transfected Vpu and tetherin plasmids was 2∶1, unless otherwise indicated.

### Viral production and infectivity assay

HIV-1 particles were produced by transient transfection of 293T cells with CaCl2 or Fugene (Roche). Unless otherwise indicated, the supernatant of producer cells was collected 36 hours post-transfection. Virion release was scored by monitoring the reverse transcriptase enzymatic activity in the producer cells supernatant. In single-round infectivity assays, viral titer was determined by applying filtered supernatant from producer cells on HeLa-CD4-LTR-LacZ indicator cells [59]. When Vpu and tetherin were co-transfected with a proviral construct, the plasmid molar ratio was 2∶2∶1, respectively, unless otherwise indicated. When required, statistical analysis of the results were performed with the InStat software (GraphPad).

### Protein analysis

Unless otherwise indicated, cells were lysed with RIPA buffer 36 hours post-transfection. Lysates were pre-cleared (13'000 rpm tabletop spin for 10 minutes), and subjected to standard SDS-PAGE, after protein quantification with the BCA kit (Thermo). Overexpressed tetherin was detected with antibodies against the relevant tag added on its N-terminus. Namely, the HA and Flag tags were detected with the mouse monoclonal antibodies 3F10 (Roche) and M2 (Sigma), respectively. The endogenous tetherin and the Vpu protein were detected with rabbit anti-tetherin and anti-Vpu antibodies, respectively, both made by K. Strebel [52],[60] (obtained through the AIDS Research and Reference Reagent Program, Division of AIDS, NIAID, NIH). All western blots of endogenous or tagged tetherin depict its glycosylated forms in the 28 to 37 kDa range, but not its immature 20 kDa form. Depending on the experiments, the relative intensity of individual tetherin bands in the 28–37 Kd range varies and we always depict the predominant species. PCNA (Oncogene Research Products) and GFP (Miltenyi) antibodies were of mouse origin, while anti-ezrin (Cell Signaling Technology) was raised in rabbits. RIG-I and ubiquitin were detected with the mouse monoclonal antibodies Alme-1 (Alexis Biochemicals) and FK-2 (BioMol International), respectively. Gag p55 and p24 were detected with the mouse monoclonal antibody made by Bruce Chesebro and Kathy Wehrly [61] (obtained through the AIDS Research and Reference Reagent Program, Division of AIDS, NIAID, NIH). Quantifications of tetherin protein levels were performed by densitometry using Photoshop (Adobe), with normalization for loading input by the parallel quantification of a control cellular protein. When required, statistical analysis of the results were performed with the InStat software (GraphPad).

### Immunoprecipitation

Lysates were prepared as described for the western blotting protein analysis. HA-tetherin was immunoprecipitated overnight in PBS, using anti-HA affinity matrix (clone 3F10, Roche Applied Science). The resulting immunoprecipitates were washed three times with RIPA buffer. They were then resuspended in Laemmli sample buffer, followed by western blot analysis.

### RNA interference

To achieve downregulation of the VCP (p97) mRNA, 293T cells were transfected using HiPerFect (Qiagen) with 100 nM of either a siRNA pool specific for this RNA (“siRNA ON-Target plus smart pool”, # L-008727-00, from Dharmacon), or a non-targeting siRNA (“Dharmacon siGenome Non-Targeting siRNA”). Twenty-four hours later, the cells were split into the adequate number of wells, and transfected with the plasmids indicated in the relevant figure.

The pGIPZ lentiviral vectors expressing, under the control of a CMV promoter, the shRNAmirs specific for β-TrCP1 or β-TrCP2 were obtained from Open Biosystems. The targeted sequences were: β-TrCP1 shRNAmir #325 (GGCACATAAACTCGTATCTTAA), β-TrCP2 shRNAmir #187 (TGCCAATTATCTGTTTGAAATA), β-TrCP2 shRNAmir #190 (GACATATTAACTCTTACCTGAA) β-TrCP2 shRNAmir #192 (GGCCTACGAGATAATTCTATTA). The production of the lentiviral vector particles serving for the delivery of these shRNAmirs were done according to the manufacturer instruction (which is a standard procedure). The transduced cells were selected with puromycin to generate stable cell lines.

### Real-time RT-PCR

Total RNA was extracted from cells with the help of the RNeasy mini kit (Qiagen), including an on-column DNase treatment step. The integrity of the resulting RNAs was checked with a spectrophotometer. Then, they served as templates for the synthesis of cDNA by the Superscript II reverse transcriptase kit (Invitrogen), using random primers. The cDNAs were quantified by SYBR-green-based real-time PCR using JumpStart SYBR green Taq ReadyMix (Sigma), on a CFX96 cycler (Bio-Rad), with the following primers: β-TrCP1 (sense CCAACATGGGCACATAAACTCG, antisense GCAGCACATAGTGATTTGGCATCC), β-TrCP2 (sense ACGAATGGTACGCACTGATCC, antisense ACTTCACCCGTGTTCACATCC), tetherin (sense CTGCAACCACACTGTGATG, antisense ACGCGTCCTGAAGCTTATG), TBP (sense GCCCGAAACGCCGAATATA, antisense: CGTGGCTCTCTTATCCTCATGA), p97 (sense: TTGCTCCAGACACAGTGATCC, antisense: GCCACCAATGTCATCATACCC). The TBP quantification allowed normalization for the starting amount of RNA.

### Accession numbers

The human and murine tetherin clones used in this study correspond to Swiss-Prot entries Q10589 and Q8R2Q8, respectively.
